# Supplementary material for: Combined Immunotherapy with Chemotherapy versus Bevacizumab with Chemotherapy in First-Line Treatment of Driver-Gene-Negative Non-Squamous Non-Small Cell Lung Cancer: An Updated Systematic Review and Network Meta-Analysis
Source: J Clin Med. 2022 Mar 16;11(6):1655. doi: 10.3390/jcm11061655 (PMC8956069; doi:10.3390/jcm11061655)
Supplement: Supplementary file 1 [file jcm-11-01655-s001.zip › Supplemental Figure S2.pdf]

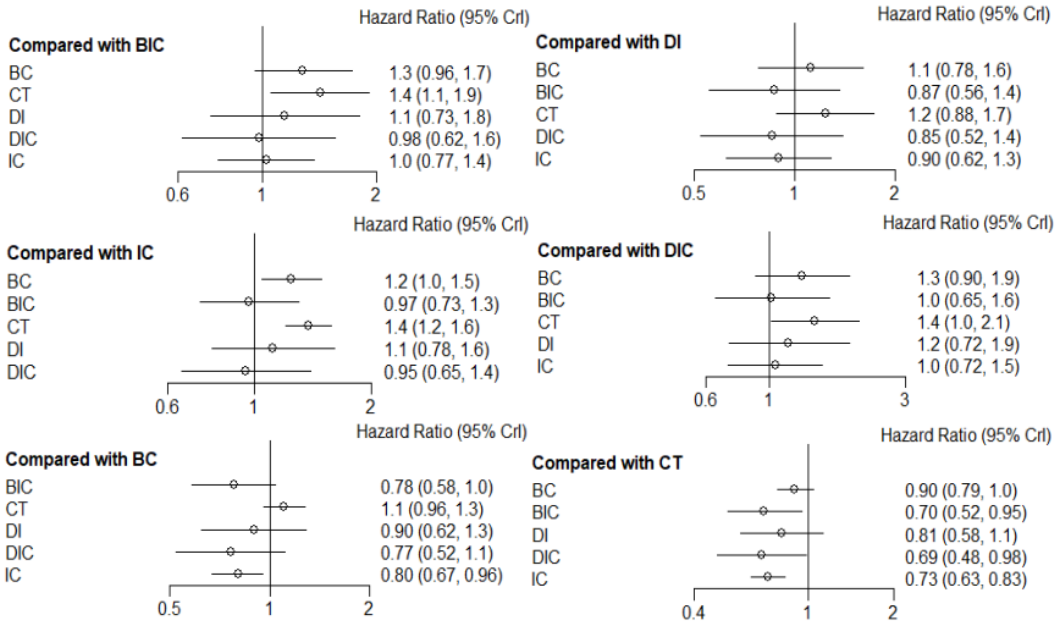

Supplemental Figure S2. Forest plot of hazard ratios (HRs) for OS in NMA.

Abbreviation: CrI: confidence interval
