# Supplementary material for: Combined Immunotherapy with Chemotherapy versus Bevacizumab with Chemotherapy in First-Line Treatment of Driver-Gene-Negative Non-Squamous Non-Small Cell Lung Cancer: An Updated Systematic Review and Network Meta-Analysis
Source: J Clin Med. 2022 Mar 16;11(6):1655. doi: 10.3390/jcm11061655 (PMC8956069; doi:10.3390/jcm11061655)
Supplement: Supplementary file 1 [file jcm-11-01655-s001.zip › Supplemental Table S1.pdf]

Supplemental Table S1. Search Strategy

Non-squamous non small cell lung cancer	#1	((((''non-squamous''[Title/Abstract]) AND ''lung cancer''[Title/Abstract]) AND (((((NSCLC[Title/Abstract]) OR "Non Small Cell"[Title/Abstract]) OR "Non-Small-Cell"[Title/Abstract]) OR "Non-Small Cell"[Title/Abstract]))) OR "Carcinoma, Non-Small-Cell Lung"[Mesh])
Immunotherapy	#2	(("pembrolizumab" [Supplementary Concept] OR "lambrolizumab" [Title/Abstract] OR "Keytruda" [Title/Abstract] OR "MK-3475" [Title/Abstract] OR "nivolumab" [Supplementary Concept] OR "MDX-1106" [Title/Abstract] OR "ONO-4538" [Title/Abstract] OR "BMS-936558" [Title/Abstract] OR "Opdivo"[Title/Abstract] OR "atezolizumab"[Supplementary Concept] OR "MPDL3280A"[Title/Abstract] OR "Tecentriq"[Title/Abstract] OR"RG7446"[Title/Abstract] OR "RG-7446"[Title/Abstract] OR "Durvalumab" [Title/Abstract] OR "Imfinzi" [Title/Abstract] OR " MEDI4736" [Title/Abstract] OR "Camrelizumab" [Title/Abstract] OR "SHR-1210" [Title/Abstract] OR " Tislelizumab" [Title/Abstract] OR "BGB-A317"[Title/Abstract] OR "Sintilimab" [Title/Abstract] OR "anti-PDL1"[Title/Abstract] OR "anti-PD1"[Title/Abstract] OR "PD-1"[Title/Abstract] OR "PD-L1"[Title/Abstract]OR "Programmed Death 1"[Title/Abstract] OR "Programmed Cell Death 1 Receptor"[Title/Abstract] OR "Programmed Death-Ligand 1"[Title/Abstract] OR "programmed cell death 1 ligand 1 protein"[Title/Abstract] OR "immune checkpoint inhibitor"[Title/Abstract] OR "immune therapy"[Title/Abstract] OR "immunotherapy"[Title/Abstract])) OR ("CTLA 4 Antigen" [Title/Abstract] OR "Cytotoxic T Lymphocyte Associated Antigen 4" [Title/Abstract] OR "CD152 Antigen*" [Title/Abstract] OR "CTLA-4 Protein" [Title/Abstract] OR "Cytotoxic T Lymphocyte Antigen 4" [Title/Abstract] OR Nivolumab[Title/Abstract] OR ONO-4538[Title/Abstract] OR MDX-1106[Title/Abstract] OR BMS-936558[Title/Abstract] OR Ipilimumab[Title/Abstract] OR MDX-010[Title/Abstract] OR Tremelimumab[Title/Abstract] OR CP-675[Title/Abstract])
Angiogenesis Inhibitors	#3	(("Bevacizumab"[Title/Abstract] OR "Avastin"[Title/Abstract])) OR "Endostatins" [Title/Abstract] OR ("Anlotinib" [Title/Abstract] OR "AL3818" [Title/Abstract]) OR "Nintedanib esylate"[Title/Abstract] OR "Ofev" [Title/Abstract] OR "nintedanib"[Title/Abstract] OR "Vargatef" [Title/Abstract] OR "BIBF 1120" [Title/Abstract] OR "BIBF-1120" [Title/Abstract] OR "Angiogenesis Inhibitor*"
Type of Trials	#4	((“clinical trials as topic"[MeSH Terms] OR “Randomized clinical trial”[Title/Abstract] OR phase[Title/Abstract]))
Date of publication	#5	2000/01/01:2021/12/31[Date - Publication]
Language	#6	"English"[Language]
Search strategy	#1 AND(#2 OR #3) AND #4 AND #5 AND #6	
