# Supplementary material for: Combined Immunotherapy with Chemotherapy versus Bevacizumab with Chemotherapy in First-Line Treatment of Driver-Gene-Negative Non-Squamous Non-Small Cell Lung Cancer: An Updated Systematic Review and Network Meta-Analysis
Source: J Clin Med. 2022 Mar 16;11(6):1655. doi: 10.3390/jcm11061655 (PMC8956069; doi:10.3390/jcm11061655)
Supplement: Supplementary file 1 [file jcm-11-01655-s001.zip › Supplemental Table S2.pdf]

**Supplemental Table S2**: Rank probabilities with SUCRA value for different outcomes in 6 kinds of first-line treatments for patients with non-squamous NSCLC.

| **OS** | | | | | | | |
|---|---|---|---|---|---|---|---|
| Treatment | Rank1 | Rank2 | Rank3 | Rank4 | Rank5 | Rank6 | SUCRA |
| IC | 0.14 | 0.38 | 0.37 | 0.11 | 0.00 | 0.00 | 0.7079 |
| BC | 0.00 | 0.00 | 0.04 | 0.27 | 0.62 | 0.06 | 0.2615 |
| BIC | 0.33 | 0.29 | 0.22 | 0.12 | 0.02 | 0.01 | 0.7506 |
| DIC | 0.44 | 0.20 | 0.18 | 0.12 | 0.04 | 0.02 | **0.7641** |
| DI | 0.09 | 0.13 | 0.18 | 0.35 | 0.16 | 0.09 | 0.4766 |
| CT | 0.00 | 0.00 | 0.00 | 0.02 | 0.16 | 0.82 | 0.0394 |
| **PFS** | | | | | | | |
| Treatment | Rank1 | Rank2 | Rank3 | Rank4 | Rank5 | Rank6 | SUCRA |
| IC | 0.27 | 0.59 | 0.13 | 0.01 | 0.00 | 0.00 | 0.8248 |
| BC | 0.00 | 0.00 | 0.13 | 0.41 | 0.40 | 0.05 | 0.3286 |
| BIC | 0.63 | 0.22 | 0.10 | 0.03 | 0.00 | 0.00 | **0.8851** |
| DIC | 0.07 | 0.12 | 0.40 | 0.21 | 0.12 | 0.07 | 0.5216 |
| DI | 0.03 | 0.06 | 0.23 | 0.29 | 0.23 | 0.16 | 0.3732 |
| CT | 0.00 | 0.00 | 0.00 | 0.04 | 0.25 | 0.71 | 0.0666 |
| **ORR** | | | | | | | |
| Treatment | Rank1 | Rank2 | Rank3 | Rank4 | Rank5 | Rank6 | SUCRA |
| IC | 0.03 | 0.29 | 0.45 | 0.22 | 0.01 | 0.00 | 0.6250 |
| BC | 0.00 | 0.45 | 0.37 | 0.16 | 0.00 | 0.00 | 0.6590 |
| BIC | 0.91 | 0.07 | 0.02 | 0.00 | 0.00 | 0.00 | **0.9760** |
| DIC | 0.05 | 0.18 | 0.15 | 0.49 | 0.12 | 0.00 | 0.5020 |
| DI | 0.00 | 0.01 | 0.01 | 0.12 | 0.62 | 0.23 | 0.1880 |
| CT | 0.00 | 0.00 | 0.00 | 0.00 | 0.24 | 0.75 | 0.0510 |
| **≥ 3 TRAEs** | | | | | | | |
| Treatment | Rank1 | Rank2 | Rank3 | - | - | - | SUCRA |
| IC | 0.27 | 0.71 | 0.02 | - | - | - | 0.6230 |
| BC | 0.73 | 0.26 | 0.01 | - | - | - | **0.8600** |
| BIC | - | - | - | - | - | - | - |
| DIC | - | - | - | - | - | - | - |
| DI | - | - | - | - | - | - | - |
| CT | 0.00 | 0.03 | 0.96 | - | - | - | 0.0017 |

***Abbreviations*****:** NSCLC, non-small cell lung cancer, ORR, objective response rate; PFS, progression-free survival; OS, overall survival; TRAE: treatment-related adverse event (grade3-5): SUCRA. surface under the cumulative ranking curve.
