# Supplementary material for: Combined Immunotherapy with Chemotherapy versus Bevacizumab with Chemotherapy in First-Line Treatment of Driver-Gene-Negative Non-Squamous Non-Small Cell Lung Cancer: An Updated Systematic Review and Network Meta-Analysis
Source: J Clin Med. 2022 Mar 16;11(6):1655. doi: 10.3390/jcm11061655 (PMC8956069; doi:10.3390/jcm11061655)
Supplement: Supplementary file 1 [file jcm-11-01655-s001.zip › Supplemental Table S3.pdf]

Supplemental Table S3: Rank probabilities with SUCRA value for different outcomes in 6 kinds of first-line treatments for subgroup patients with non-squamous NSCLC.

**OS of patients with ECOG = 0**							
Treatment	Rank1	Rank2	Rank3	Rank4	-	-	SUCRA
IC	0.21	0.30	0.25	0.23	-	-	0.4969
BC	0.04	0.42	0.35	0.19	-	-	0.4353
BIC	0.73	0.17	0.06	0.05	-	-	**0.8695**
DIC	-	-	-	-	-	-	-
DI	-	-	-	-	-	-	-
CT	0.02	0.11	0.34	0.53	-	-	0.2083
**PFS of patients with ECOG = 0**							
Treatment	Rank1	Rank2	Rank3	-	-	-	SUCRA
IC	0.19	0.81	0.00	-	-	-	0.5944
BC	0.81	0.19	0.00	-	-	-	**0.9030**
BIC	-	-	-	-	-	-	-
DIC	-	-	-	-	-	-	-
DI	-	-	-	-	-	-	-
CT	0.00	0.00	0.99	-	-	-	0.0025
**OS of patients with ECOG = 1**							
Treatment	Rank1	Rank2	Rank3	Rank4	-	-	SUCRA
IC	0.25	0.19	0.36	0.20	-	-	0.4962
BC	0.23	0.50	0.23	0.03	-	-	0.6415
BIC	0.51	0.25	0.14	0.11	-	-	**0.7176**
DIC	-	-	-	-	-	-	-
DI	-	-	-	-	-	-	-
CT	0.01	0.06	0.27	0.65	-	-	0.1447
**PFS of patients with ECOG = 1**							
Treatment	Rank1	Rank2	Rank3	-	-	-	SUCRA
IC	0.04	0.96	0.00	-	-	-	0.5207
BC	0.96	0.04	0.00	-	-	-	**0.9783**
BIC	-	-	-	-	-	-	-
DIC	-	-	-	-	-	-	-
DI	-	-	-	-	-	-	-
CT	0.00	0.00	1.00	-	-	-	0.0010
**OS of smokers**							
Treatment	Rank1	Rank2	Rank3	Rank4	-	-	SUCRA
IC	0.22	0.21	0.46	0.11	-	-	0.5145
BC	0.09	0.53	0.27	0.11	-	-	0.5287
BIC	0.68	0.20	0.08	0.04	-	-	**0.8407**
DIC	-	-	-	-	-	-	-
DI	-	-	-	-	-	-	-
CT	0.01	0.07	0.19	0.73	-	-	0.1162

**PFS of smokers**							
Treatment	Rank1	Rank2	Rank3	-	-	-	SUCRA
IC	0.31	0.69	0.00	-	-	-	0.6526
BC	0.69	0.30	0.01	-	-	-	**0.8443**
BIC	-	-	-	-	-	-	-
DIC	-	-	-	-	-	-	-
DI	-	-	-	-	-	-	-
CT	0.00	0.01	0.99	-	-	-	0.0032
**OS of non-smokers**							
Treatment	Rank1	Rank2	Rank3	Rank4	-	-	SUCRA
IC	0.32	0.21	0.35	0.11	-	-	0.5830
BC	0.11	0.47	0.37	0.05	-	-	0.5479
BIC	0.56	0.28	0.11	0.05	-	-	**0.7830**
DIC	-	-	-	-	-	-	-
DI	-	-	-	-	-	-	-
CT	0.01	0.04	0.16	0.79	-	-	0.0860
**PFS of non-smokers**							
Treatment	Rank1	Rank2	Rank3	-	-	-	SUCRA
IC	0.09	0.88	0.03	-	-	-	0.5262
BC	0.91	0.08	0.00	-	-	-	**0.9522**
BIC	-	-	-	-	-	-	-
DIC	-	-	-	-	-	-	-
DI	-	-	-	-	-	-	-
CT	0.00	0.04	0.96	-	-	-	0.0216
**OS of patients ≥ 65 years old**							
Treatment	Rank1	Rank2	Rank3	-	-	-	SUCRA
IC	0.44	0.43	0.13	-	-	-	0.6569
BC	0.54	0.39	0.06	-	-	-	**0.7396**
BIC	-	-	-	-	-	-	-
DIC	-	-	-	-	-	-	-
DI	-	-	-	-	-	-	-
CT	0.01	0.18	0.81	-	-	-	0.1035
**PFS of patients ≥ 65 years old**							
Treatment	Rank1	Rank2	Rank3	-	-	-	SUCRA
IC	0.44	0.43	0.13	-	-	-	0.6538
BC	0.55	0.39	0.06	-	-	-	**0.7434**
BIC	-	-	-	-	-	-	-
DIC	-	-	-	-	-	-	-
DI	-	-	-	-	-	-	-
CT	0.01	0.18	0.81	-	-	-	0.1028
**OS of patients < 65 years old**							
Treatment	Rank1	Rank2	Rank3	Rank4	-	-	SUCRA

IC	0.24	0.23	0.33	0.20	-	-	0.5034
BC	0.12	0.50	0.33	0.06	-	-	0.5591
BIC	0.64	0.21	0.09	0.07	-	-	**0.8067**
DIC	-	-	-	-	-	-	-
DI	-	-	-	-	-	-	-
CT	0.01	0.05	0.25	0.68	-	-	0.1308
**PFS of patients < 65 years old**							
Treatment	Rank1	Rank2	Rank3	-	-	-	SUCRA
IC	0.02	0.98	0.00	-	-	-	0.5076
BC	0.98	0.02	0.00	-	-	-	**0.9912**
BIC	-	-	-	-	-	-	-
DIC	-	-	-	-	-	-	-
DI	-	-	-	-	-	-	-
CT	0.00	0.00	1.00	-	-	-	0.0012
**OS of male patients**							
Treatment	Rank1	Rank2	Rank3	Rank4	-	-	SUCRA
IC	0.07	0.13	0.55	0.25	-	-	0.3398
BC	0.08	0.75	0.15	0.01	-	-	0.6352
BIC	0.85	0.10	0.03	0.01	-	-	**0.9269**
DIC	-	-	-	-	-	-	-
DI	-	-	-	-	-	-	-
CT	0.00	0.01	0.26	0.72	-	-	0.0981
**PFS of male patients**							
Treatment	Rank1	Rank2	Rank3	-	-	-	SUCRA
IC	0.10	0.90	0.00	-	-	-	0.5470
BC	0.90	0.09	0.00	-	-	-	**0.9502**
BIC	-	-	-	-	-	-	-
DIC	-	-	-	-	-	-	-
DI	-	-	-	-	-	-	-
CT	0.00	0.01	0.99	-	-	-	0.0028
**OS of female patients**							
Treatment	Rank1	Rank2	Rank3	Rank4	-	-	SUCRA
IC	0.53	0.24	0.16	0.08	-	-	**0.7356**
BC	0.07	0.31	0.48	0.13	-	-	0.4398
BIC	0.39	0.36	0.13	0.12	-	-	0.6748
DIC	-	-	-	-	-	-	-
DI	-	-	-	-	-	-	-
CT	0.01	0.09	0.23	0.66	-	-	0.1497
**PFS of female patients**							
Treatment	Rank1	Rank2	Rank3	-	-	-	SUCRA
IC	0.16	0.84	0.00	-	-	-	0.5799
BC	0.84	0.16	0.00	-	-	-	**0.9177**

BIC	-	-	-	-	-	-	-
DIC	-	-	-	-	-	-	-
DI	-	-	-	-	-	-	-
CT	0.00	0.00	1.00	-	-	-	0.0023
**OS of patients with liver metastases**							
Treatment	Rank1	Rank2	Rank3	-	-	-	SUCRA
IC	0.32	0.52	0.17	-	-	-	0.5754
BC	0.66	0.25	0.09	-	-	-	**0.7869**
BIC	-	-	-	-	-	-	-
DIC	-	-	-	-	-	-	-
DI	-	-	-	-	-	-	-
CT	0.02	0.23	0.75	-	-	-	0.1377
**PFS of patients with liver metastases**							
Treatment	Rank1	Rank2	-	-	-	-	SUCRA
IC	0.9569	0.0431	-	-	-	-	0.9569
BC	-	-	-	-	-	-	-
BIC	-	-	-	-	-	-	-
DIC	-	-	-	-	-	-	-
DI	-	-	-	-	-	-	-
CT	0.0431	0.9569	1-	-	-	-	0.0431

Abbreviations**:** NSCLC, non-small cell lung cancer, PFS, progression-free survival; OS, overall survival; SUCRA. surface under the cumulative ranking curve.
