# Supplementary figures and images for: Combined Immunotherapy with Chemotherapy versus Bevacizumab with Chemotherapy in First-Line Treatment of Driver-Gene-Negative Non-Squamous Non-Small Cell Lung Cancer: An Updated Systematic Review and Network Meta-Analysis
Source: J Clin Med. 2022 Mar 16;11(6):1655. doi: 10.3390/jcm11061655 (PMC8956069; doi:10.3390/jcm11061655)

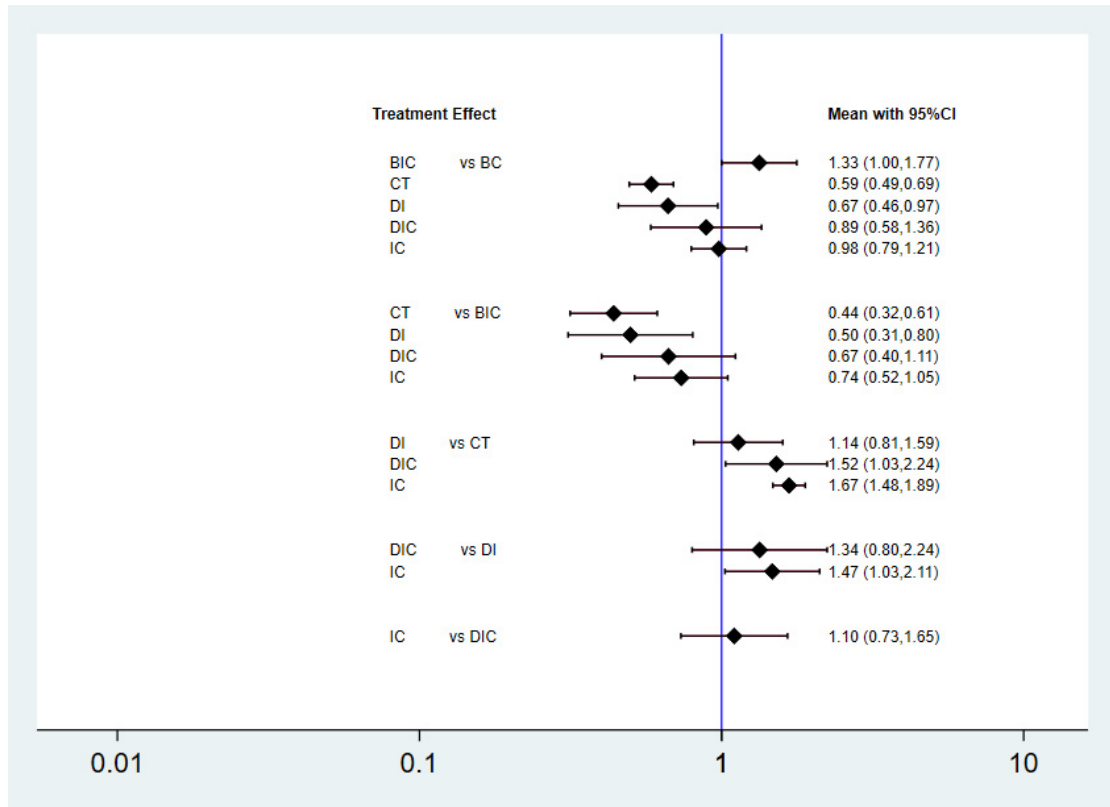

Supplemental Figure S4. Forest plot of risk ratios (RRs) for ORR in NMA.

Supplement: Supplementary file 1 [file jcm-11-01655-s001.zip › Supplemental Figure S4.pdf]

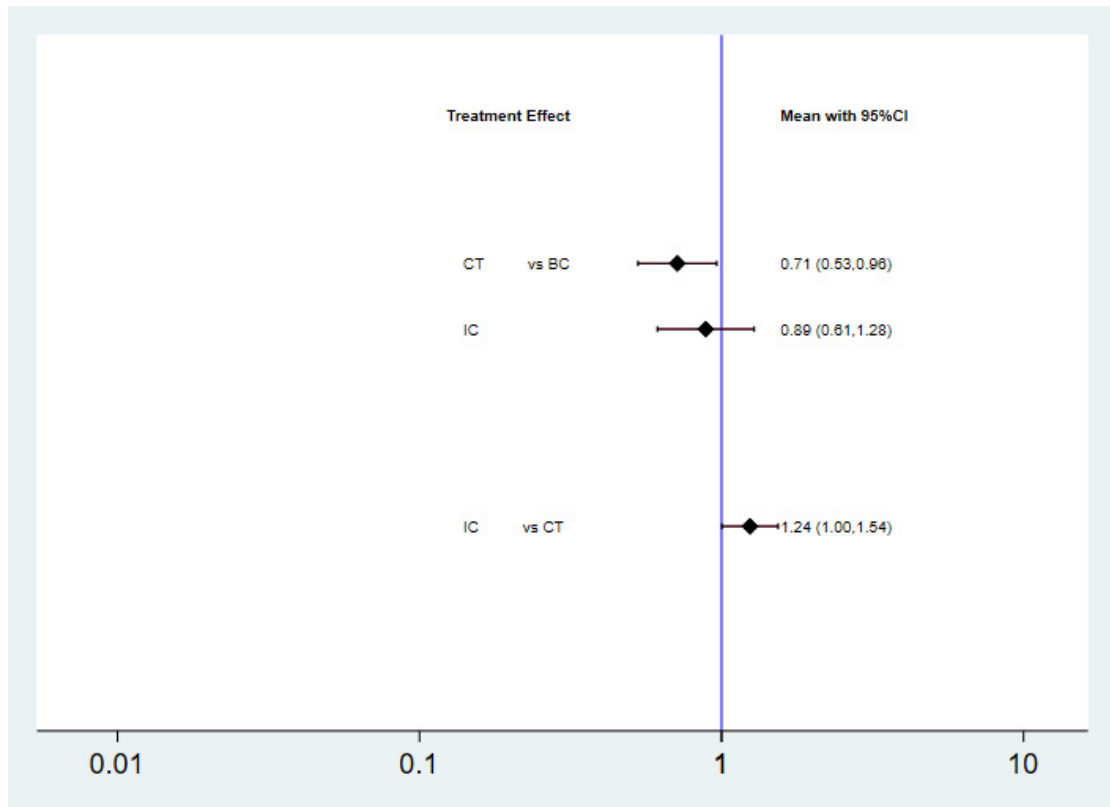

Supplemental Figure S5. Forest plot of risk ratios (RRs) for  $\geq 3$  TRAEs in NMA.

Supplement: Supplementary file 1 [file jcm-11-01655-s001.zip › Supplemental Figure S5.pdf]
